# Disease-predominant loci across Alzheimer’s disease, Parkinson’s disease and Lewy body dementia: evidence from the UK Biobank prospective cohort, conditional GWAS and colocalization

**DOI:** 10.3389/fgene.2026.1865257

**Published:** 2026-07-02

**Authors:** Ying Zhang, Zhishuai Zhang, Shizheng Qiu, Yang Hu

**Affiliations:** 1 Department of Pharmacy, Beidahuang Industry Group General Hospital, Harbin, China; 2 Center for Bioinformatics, Faculty of Computing, Harbin Institute of Technology, Harbin, China

**Keywords:** Alzheimer’s disease, colocalization, conditional GWAS, Lewy body dementia, mtCOJO, Parkinson’s disease, UK Biobank

## Abstract

**Background:**

Alzheimer’s disease (AD), Parkinson’s disease (PD) and Lewy body dementia (LBD) overlap clinically, pathologically and genetically, complicating interpretation of cross-disorder genome-wide association study (GWAS) signals.

**Methods:**

We analysed 322,963 UK Biobank participants with bidirectional time-varying Cox models, one-year and two-year lag analyses, and competing-risk sensitivity models to quantify AD-PD clinical co-occurrence. We then analysed European-ancestry AD, PD and LBD GWAS summary statistics using linkage disequilibrium score regression (LDSC), GCTA-mtCOJO/GSMR, MAGMA, stratified LDSC, brain eQTL/mQTL SMR with HEIDI filtering, and Bayesian colocalization for selected methylation probes. Conditional loci were compared with original GWAS loci to separate shared liability from retained disorder-predominant associations.

**Results:**

PD was associated with subsequent AD (fully adjusted HR 2.27, 95% CI 1.94–2.65; P = 6.40E-25), and AD was associated with subsequent PD (HR 3.14, 95% CI 2.56–3.85; P = 2.10E-28). Lag and competing-risk sensitivity analyses remained concordant. LDSC estimated positive genetic correlations for AD-PD (rg = 0.20; P = 0.0086) and PD-LBD (rg = 0.61; P = 0.0005). Conditioning reduced genome-wide significant loci from 14 to 9 for AD, from 24 to 21 for PD and from 5 to 2 for LBD. Retained loci included AD signals near CR1, BIN1, CLU, SPI1, MS4A, PICALM, ABCA7 and APOE; PD signals near GBA, NUCKS1, TMEM163, STK39, GAK/TMEM175, BST1, SNCA, LRRK2, MAPT and RIT2; and LBD signals near SNCA/MMRN1 and APOE. MAGMA and S-LDSC highlighted amyloid, lipid, immune, synaptic-vesicle and brain-tissue enrichment patterns. Brain QTL analyses prioritized retained eQTL and mQTL signals, and colocalization supported shared PD-GWAS/mQTL signals at HLA-DRB5, ARHGAP27, CRHR1, MAPT and KANSL1.

**Conclusion:**

AD and PD show bidirectional clinical co-occurrence, whereas conditional genetic analyses retain a smaller set of disease-predominant loci and regulatory signals across AD, PD and LBD. These findings refine cross-disorder interpretation and nominate loci for independent genetic and functional validation.

## Introduction

Alzheimer’s disease (AD), Parkinson’s disease (PD) and Lewy body dementia (LBD) are major neurodegenerative disorders with substantial overlap in clinical manifestations, neuropathology and inherited susceptibility (Compta et al., 2011; Guerreiro et al., 2018; Guerreiro et al., 2016; Jellinger and Korczyn, 2018; Perl et al., 1998; Iseki et al., 1999; Guo et al., 2022). LBD occupies a clinically important interface because cognitive fluctuations, visual hallucinations, parkinsonism and Lewy body pathology can coexist with AD-type amyloid plaques and neurofibrillary tangles (Matar et al., 2020; Taylor et al., 2020; O'Dowd et al., 2019; Armstrong et al., 2005; Forstl, 1999). These overlapping features can complicate distinction among LBD, Parkinson’s disease dementia and AD-related dementia in clinical and genetic studies.

Genome-wide association studies have identified susceptibility loci for AD, PD and LBD and have also reported cross-disorder genetic overlap (Guerreiro et al., 2018; Guerreiro et al., 2016; Guo et al., 2022; Kunkle et al., 2019; Chang et al., 2017; Nalls et al., 2014; Chia et al., 2021; Nalls et al., 2019; Lambert et al., 2013; Brainstorm, 2018). APOE is strongly linked to AD and LBD, GBA and SNCA-region biology connect PD and LBD, and MAPT-region variation has been implicated in overlapping neurodegenerative phenotypes (Guerreiro et al., 2018; Kunkle et al., 2019; Chang et al., 2017; Nalls et al., 2014; Chia et al., 2021; Nalls et al., 2019; Lambert et al., 2013). However, a locus observed in more than one marginal GWAS can reflect genuinely shared liability, linkage disequilibrium, mixed pathology, phenotype heterogeneity, sample structure or disorder-predominant effects that persist after accounting for related traits.

Distinguishing shared from conditionally retained signals is therefore important for biological interpretation. This issue is particularly relevant for LBD because its available GWAS sample size is smaller than that of AD and PD. It is also relevant for molecular QTL integration, where nearest-gene annotation does not necessarily identify the transcript or methylation probe most consistent with the disease association.

We combined population-level clinical timing with cross-disorder conditional genetics. First, we quantified bidirectional AD-PD clinical co-occurrence in the UK Biobank. Second, we estimated pairwise genetic correlations and used mtCOJO to identify AD, PD and LBD loci that remained genome-wide significant after conditioning on the other two disorders. Third, we integrated retained loci with MAGMA, S-LDSC, SMR/HEIDI and Bayesian colocalization to prioritize regulatory signals while avoiding causal overinterpretation.

## Methods

### UK Biobank cohort and clinical modelling

UK Biobank recruited approximately 500,000 adults aged 40–69 years from 22 assessment centres in England, Scotland and Wales between 2006 and 2010 (Allen et al., 2024; Bycroft et al., 2018; Sudlow et al., 2015). Baseline date was defined as assessment-centre attendance (Field 53), and death information was obtained from registry linkage (Field 40000). AD and PD dates were derived from first-occurrence outcome fields for Alzheimer’s disease (Field 131036; ICD-10 G30) and Parkinson’s disease (Field 131022; ICD-10 G20) (Supplementary Table S1).

The analytic cohort included participants with AD, PD, both diagnoses and neurologically healthy controls. Neurologically healthy controls had no study-defined AD, PD or other recorded neurological disorders in the phenotype extraction (Supplementary Table S2). Follow-up was censored at death or 30 September 2025, whichever occurred first.

Bidirectional clinical co-occurrence was evaluated with time-varying Cox regression using time since baseline as the analysis time scale. In the PD-to-AD analysis, PD was modelled as a time-updated exposure and incident AD as the outcome; participants with AD at or before baseline were excluded. In the AD-to-PD analysis, AD was modelled as a time-updated exposure and incident PD as the outcome; participants with PD at or before baseline were excluded. Same-day post-baseline AD and PD diagnoses were excluded from the corresponding directional analysis.

Model 1 adjusted for age, sex and race. Model 2 additionally adjusted for current smoking, current drinking, higher education, body mass index and total cholesterol. Reverse-causation sensitivity analyses excluded outcomes occurring within one and 2 years after exposure onset. Death was evaluated as a competing event in subdistribution-hazard sensitivity models in which competing deaths remained in the risk set after death and the time-updated exposure was frozen at death.

### GWAS resources and conditional genetic analysis

AD summary statistics came from the International Genomics of Alzheimer’s Project meta-analysis of 21,982 clinically diagnosed or autopsy-documented AD cases and 41,944 controls (Kunkle et al., 2019). PD summary statistics came from the International Parkinson’s Disease Genomics Consortium meta-analysis of 33,674 clinician-ascertained PD cases and 449,056 controls (Nalls et al., 2019). LBD summary statistics came from 2,981 LBD cases, including 1,789 autopsy-confirmed and 802 clinically diagnosed cases, and 2,173 neurologically healthy controls (Chia et al., 2021) (Table 1; Supplementary Table S1).

**TABLE 1:** Source GWAS summary statistics and conditional GWAS retention.

Trait	Source	Cases	Controls	Ancestry	Original lead SNPs (loci)	Conditional lead SNPs (loci)	Newly prioritized loci after conditioning
AD	IGAP	21982	41944	European	77 (14)	56 (9)	1
PD	IPDGC	33674	449056	European	32 (24)	29 (21)	5
LBD	Chia et al.	2981	2173	European/North American of European ancestry	10 (5)	3 (2)	1

AD, Alzheimer’s disease; PD, Parkinson’s disease; LBD, Lewy body dementia.

Pairwise genetic correlations among AD, PD and LBD were estimated with bivariate LDSC using HapMap3 SNPs and European LD scores. Genetic correlation P values were interpreted with Bonferroni correction across three trait pairs (P < 0.05/3). LDSC intercepts were used as summary-statistics checks for residual confounding and sample-overlap signal.

We used the mtCOJO framework implemented in GCTA to estimate conditional SNP effects for each disorder while accounting for the genetic effects of the other two disorders (Zhu et al., 2018). For each target disorder, the remaining two disorders were modelled as genetically correlated covariates, and GSMR estimated cross-trait effects for conditioning. Independent SNP instruments were selected after clumping against the European subset of the 1000 Genomes Project Phase III reference panel. Summary statistics were harmonized by rsID and allele orientation, and 6,732,472 autosomal SNPs retained after quality control were included in conditional analysis.

The default instrument threshold was genome-wide significance (P < 5E-08). Because the LBD GWAS yielded fewer than 10 independent genome-wide significant instruments, the LBD exposure threshold was relaxed to P < 1E-06 to permit GSMR conditioning. This threshold was used only for instrument availability. Loci retained at P < 5E-08 after conditioning are described as conditionally retained or disease-predominant rather than disease-exclusive.

### Gene-set, tissue and molecular analyses

MAGMA was used for gene-based and gene-set association testing for both original and conditional GWAS summary statistics (de Leeuw et al., 2015). GO and KEGG gene sets were evaluated, and genes or gene sets passing multiple-testing correction were treated as prioritized statistical signals.

Tissue and cell-type enrichment was evaluated using S-LDSC with specifically expressed gene annotations from 205 GTEx and Franke laboratory tissues (Finucane et al., 2018; GTEx Consortium, 2017; Pers et al., 2015; Fehrmann et al., 2015). SNP signals from original and conditional summary statistics were mapped to regions around genes with high tissue-specific expression, and the Bonferroni tissue threshold was P < 0.05/205.

Brain eQTL and mQTL summary statistics were derived from meta-analyses including ROSMAP, Braineac, CAGE, CommonMind, GTEx and eQTLGen resources (GTEx Consortium, 2017; Qi et al., 2018; Fromer et al., 2016; Ng et al., 2017; Trabzuni et al., 2011; Lloyd-Jones et al., 2017). The brain meta-eQTL resource contained 1,194 samples and 28,538 probes, and the brain meta-mQTL resource contained 1,160 samples and 436,077 probes. After GWAS-probe matching, 7,461 and 7,225 eQTL probes and 96,133 and 93,109 mQTL probes were analysed for original and conditional GWAS, respectively. SNPs with LD r-squared >0.90 or <0.05 for each probe were excluded before SMR/HEIDI testing.

SMR tested whether disease associations and molecular QTL associations were consistent with a shared genetic instrument (Zhu et al., 2016; Pavlides et al., 2016; Hannon et al., 2017). HEIDI was used to reduce linkage-driven findings; prioritized probes required Bonferroni-significant SMR evidence and HEIDI P > 0.05. These signals were interpreted as regulatory prioritization results, not proof of causality.

Bayesian colocalization was performed for selected SMR-prioritized mQTL probes with available probe-level QTL summary statistics using coloc.abf (Giambartolomei et al., 2014). Disease and mQTL summary statistics were harmonized by rsID and allele orientation. Posterior support was summarized for five hypotheses, with PP.H4 interpreted as support for a shared disease and mQTL association signal and PP.H3 interpreted as support for distinct disease and mQTL association signals within the same region.

## Results

### Bidirectional clinical co-occurrence between AD and PD

The UK Biobank analysis included 322,963 participants: 313,127 neurologically healthy controls, 4,607 participants with PD only, 4,894 with AD only and 335 with both AD and PD (Table 2). Participants with AD or PD were older than neurologically healthy controls; male sex was more frequent in the PD-only group and in participants with both diagnoses. Among participants with both diagnoses, PD preceded AD in 165 individuals, AD preceded PD in 98 and same-day diagnoses occurred in 72 (Supplementary Table S3).

**TABLE 2:** Baseline characteristics of UK Biobank participants by AD/PD status.

Characteristic	Neurologically healthy control	Parkinson disease only	Alzheimer disease only	Both Alzheimer disease and Parkinson disease
Participants	313127	4,607	4,894	335
Age	56.32 (8.10)	62.47 (5.53)	64.63 (4.27)	64.87 (3.62)
Sex: Male	146644 (46.8%)	2,877 (62.4%)	2,254 (46.1%)	216 (64.5%)
Race: White	293627 (93.8%)	4,430 (96.2%)	4,728 (96.6%)	315 (94.0%)
Current smoking	11147 (3.6%)	214 (4.6%)	336 (6.9%)	14 (4.2%)
Current drinking	291401 (93.1%)	4,081 (88.6%)	4,275 (87.4%)	285 (85.1%)
Higher education	108254 (34.6%)	1,335 (29.0%)	956 (19.5%)	82 (24.5%)
Body mass index	27.16 (4.52)	27.76 (4.49)	27.47 (4.66)	27.23 (4.45)
Total cholesterol	5.71 (1.09)	5.47 (1.14)	5.68 (1.24)	5.32 (1.17)

**FIGURE 1:**
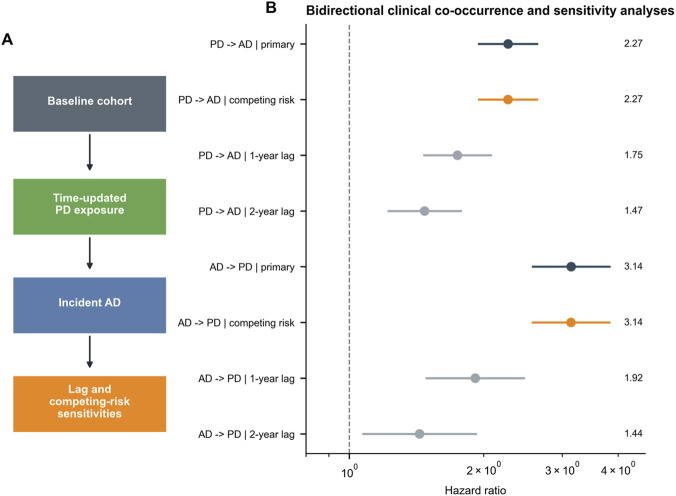
Bidirectional clinical co-occurrence between Alzheimer’s disease and Parkinson’s disease in the UK Biobank. **(A)** Time-updated modelling framework for the primary analysis and sensitivity analyses. The exposure disease was updated at its first recorded post-baseline diagnosis date, and follow-up was censored at the outcome, death or 30 September 2025. **(B)** Fully adjusted time-varying Cox estimates, competing-risk sensitivity estimates, and one-year and two-year lag sensitivity estimates. Points indicate hazard ratios and horizontal lines indicate 95% confidence intervals; numeric labels show point estimates.

In Model 1 time-varying Cox analyses, PD was associated with subsequent AD (HR 2.39, 95% CI 2.05–2.80; P = 3.90E-28), and AD was associated with subsequent PD (HR 3.43, 95% CI 2.80–4.20; P = 8.66E-33). The associations remained strong in fully adjusted Model 2: PD to subsequent AD (HR 2.27, 95% CI 1.94–2.65; P = 6.40E-25; 5,135 events) and AD to subsequent PD (HR 3.14, 95% CI 2.56–3.85; P = 2.10E-28; 3,940 events; Table 3; Figure 1). Competing-risk sensitivity estimates were nearly identical, and one-year and two-year lag analyses attenuated but did not remove the associations.

**TABLE 3:** Bidirectional AD-PD association and sensitivity analyses in the UK Biobank.

Analysis	Direction	Participants	Events	HR (95% CI)	P value
Model 1 time-varying cox	Parkinson disease to subsequent Alzheimer disease	322869	5135	2.39 (2.05–2.80)	3.90E-28
Model 2 time-varying cox	Parkinson disease to subsequent Alzheimer disease	322869	5135	2.27 (1.94–2.65)	6.40E-25
Competing-risk sensitivity	Parkinson disease to subsequent Alzheimer disease	322869	5135	2.27 (1.94–2.65)	6.76E-25
1-year lag	Parkinson disease to subsequent Alzheimer disease	322831	5097	1.75 (1.46–2.09)	5.96E-10
2-year lag	Parkinson disease to subsequent Alzheimer disease	322811	5077	1.47 (1.22–1.79)	7.37E-05
Model 1 time-varying cox	Alzheimer disease to subsequent Parkinson disease	321961	3940	3.43 (2.80–4.20)	8.66E-33
Model 2 time-varying cox	Alzheimer disease to subsequent Parkinson disease	321961	3940	3.14 (2.56–3.85)	2.10E-28
Competing-risk sensitivity	Alzheimer disease to subsequent Parkinson disease	321961	3940	3.14 (2.56–3.84)	2.33E-28
1-year lag	Alzheimer disease to subsequent Parkinson disease	321923	3902	1.92 (1.48–2.48)	7.08E-07
2-year lag	Alzheimer disease to subsequent Parkinson disease	321908	3887	1.44 (1.07–1.93)	0.016

### Shared genetic liability and conditionally retained loci

**FIGURE 2:**
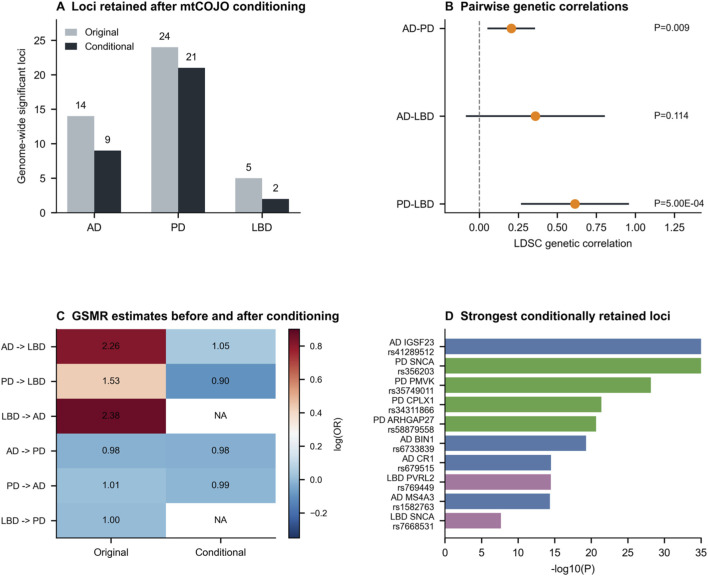
Cross-disorder genetic sharing and conditionally retained GWAS signals. **(A)** Genome-wide significant locus counts in original and mtCOJO-conditioned analyses. Bars show the number of independent genome-wide significant loci before and after conditioning on the other two disorders. **(B)** Pairwise LDSC genetic correlations among AD, PD and LBD. Points show rg estimates, horizontal lines show 95% confidence intervals and labels show LDSC P values. **(C)** GSMR cross-trait odds-ratio estimates before and after conditioning. Values are odds ratios; colour indicates log(OR). **(D)** Strongest conditionally retained loci ranked by -log10(P), with disease colours indicating the target GWAS.

The GWAS datasets differed substantially in size, with fewer LBD cases than AD or PD cases (Table 1). Conditional GWAS reduced genome-wide significant loci from 14 to 9 for AD, from 24 to 21 for PD and from 5 to 2 for LBD (Figure 2A; Supplementary Table S4). The corresponding lead-SNP counts were reduced from 77 to 56 for AD, from 32 to 29 for PD and from 10 to 3 for LBD (Table 1).

For AD, retained loci included CR1, BIN1, OARD1/APOBEC2, CLU, SPI1, MS4A, PICALM/RNU6-560P, ABCA7 and the APOE-region signal. For PD, retained loci included GBA-region/KRTCAP2, NUCKS1, TMEM163, STK39, ABHD6, MCCC1, GAK/TMEM175, BST1, SCARB2/FAM47E, SNCA, NDUFAF2, NUPL2/GPNMB, INPP5F, IGSF9B, LRRK2, CCDC62, CTF1, MAPT and RIT2-region signals. For LBD, retained loci mapped to SNCA/MMRN1 and APOE/TOMM40/APOC1 regions (Table 4; Supplementary Table S5).

**TABLE 4:** Conditionally retained genome-wide significant loci.

Trait	Lead SNP	Chromosome	Position	Gene	Conditional beta	Conditional OR	Conditional 95% CI	Conditional P
AD	rs679515	1	207750568	CR1	0.148	1.16	1.12–1.20	3.14E-15
AD	rs6733839	2	127892810	BIN1	−0.145	0.87	0.84–0.89	5.06E-20
AD	rs114812713	6	41034000	OARD1	−0.289	0.75	0.69–0.82	8.97E-11
AD	rs867230	8	27468503	CLU	−0.125	0.88	0.86–0.91	1.14E-14
AD	rs3740688	11	47380340	SPI1	−0.087	0.92	0.89–0.94	3.74E-09
AD	rs1582763	11	60021948	MS4A4E	0.120	1.13	1.10–1.16	4.40E-15
AD	rs3851179	11	85868640	RNU6-560P	−0.110	0.90	0.87–0.92	6.20E-13
AD	rs12151021	19	1050874	ABCA7	0.099	1.10	1.07–1.14	9.15E-09
AD	rs72654472	19	45414392	APOE	0.347	1.41	1.26–1.58	1.14E-09
LBD	rs7668531	4	90791819	MMRN1	−0.212	0.81	0.75–0.87	2.26E-08
LBD	rs769449	19	45410002	APOE	−0.435	0.65	0.58–0.72	3.22E-15
PD	rs35749011	1	155135036	KRTCAP2	−0.735	0.48	0.42–0.55	6.93E-29
PD	rs823116	1	205720483	NUCKS1	−0.100	0.91	0.88–0.94	5.88E-09
PD	rs57891859	2	135464616	TMEM163	0.109	1.12	1.07–1.16	9.1E-09
PD	rs4613239	2	169119609	STK39	−0.179	0.84	0.80–0.88	5.51E-13
PD	rs4488803	3	58218352	ABHD6	0.113	1.12	1.08–1.16	1.49E-08
PD	rs10513789	3	182760073	MCCC1	0.160	1.17	1.12–1.22	3.06E-13
PD	rs3775120	4	893687	GAK	0.112	1.12	1.08–1.16	2.32E-09
PD	rs4698412	4	15737348	BST1	−0.125	0.88	0.85–0.91	1.03E-13
PD	rs7695720	4	77183300	FAM47E	0.125	1.13	1.09–1.18	2.07E-09
PD	rs356203	4	90666041	SNCA	0.239	1.27	1.23–1.32	3.86E-41
PD	rs75646569	5	60345424	NDUFAF2	−0.189	0.83	0.79–0.87	1.4E-12
PD	rs858295	7	23245569	NUPL2	0.103	1.11	1.07–1.15	4.3E-09
PD	rs620490	8	16697579	Intergenic	0.117	1.12	1.08–1.17	7.6E-10
PD	rs117896,735	10	121536327	INPP5F	−0.423	0.66	0.58–0.75	2.08E-10
PD	rs510306	11	133774863	IGSF9B	0.104	1.11	1.07–1.15	8.02E-10
PD	rs1491942	12	40620808	LRRK2	−0.120	0.89	0.85–0.92	8.95E-09
PD	rs12817488	12	123296294	CCDC62	−0.104	0.90	0.87–0.93	5.49E-09
PD	rs4774417	15	61993702	Intergenic	−0.106	0.90	0.87–0.93	3.28E-08
PD	rs12934900	16	30923602	CTF1	−0.120	0.89	0.86–0.92	6.27E-11
PD	rs58879558	17	44095467	MAPT	0.238	1.27	1.21–1.33	2.13E-21
PD	rs4588066	18	40672964	RIT2	−0.105	0.90	0.87–0.93	3.22E-09

LDSC estimated positive genetic correlations for AD-PD (rg = 0.20, SE = 0.08; P = 0.0086) and PD-LBD (rg = 0.61, SE = 0.18; P = 0.0005), whereas the AD-LBD estimate was positive but imprecise (rg = 0.36, SE = 0.23; P = 0.114; Figure 2B; Supplementary Table S6). LDSC intercepts were modest in the original GWAS comparisons.

GSMR estimates were strongest for AD to LBD (OR 2.26, P = 2.75E-130), LBD to AD (OR 2.38, P = 5.15E-40) and PD to LBD (OR 1.53, P = 3.61E-14) in the original analysis. These cross-trait estimates were attenuated after conditioning (Figure 2C; Supplementary Table S7; Supplementary Figure 1), supporting partial shared liability without implying that every retained locus was biologically exclusive to one disease.

**FIGURE 3:**
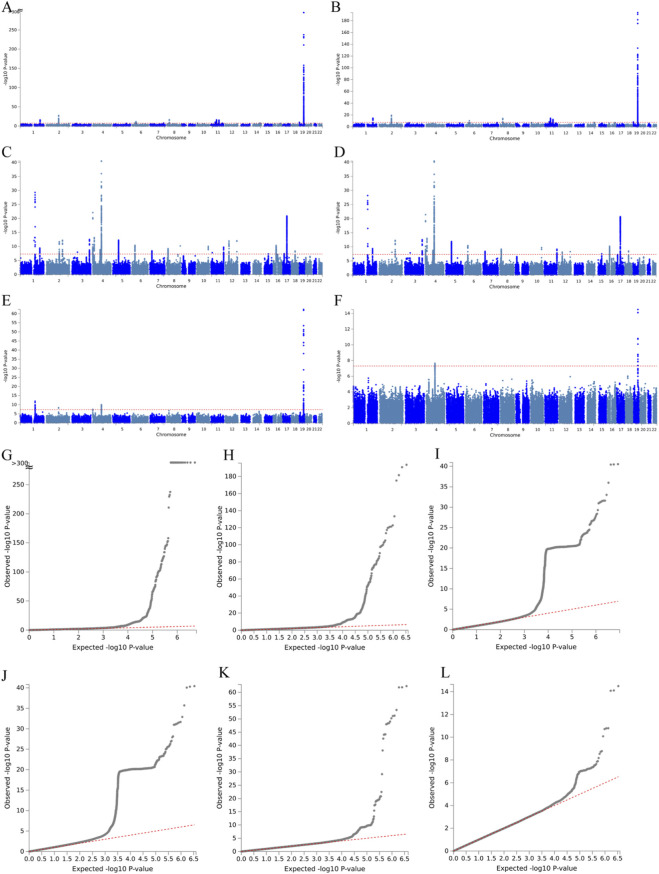
Manhattan and Q-Q plots for original and conditional GWAS analyses. Panels **(A–F)** show Manhattan plots for original AD, conditional AD, original PD, conditional PD, original LBD and conditional LBD analyses. Panels **(G–L)** show the corresponding Q-Q plots. The dashed line in Manhattan panels indicates the genome-wide significance threshold.

Most lead-SNP effects were attenuated after conditioning (Figure 2D). The APOE region remained relevant to both AD and LBD, consistent with disorder-associated variants within a shared risk region. Several conditional estimates changed direction after allele harmonization and cross-trait adjustment; these estimates were interpreted as model-derived conditional effects rather than opposite biological mechanisms. Among retained PD loci, STK39 showed a modestly stronger conditional association than in the marginal GWAS. Manhattan and Q-Q plots for original and conditional analyses are shown in Figure 3.

### Pathway, tissue and molecular prioritization

**FIGURE 4:**
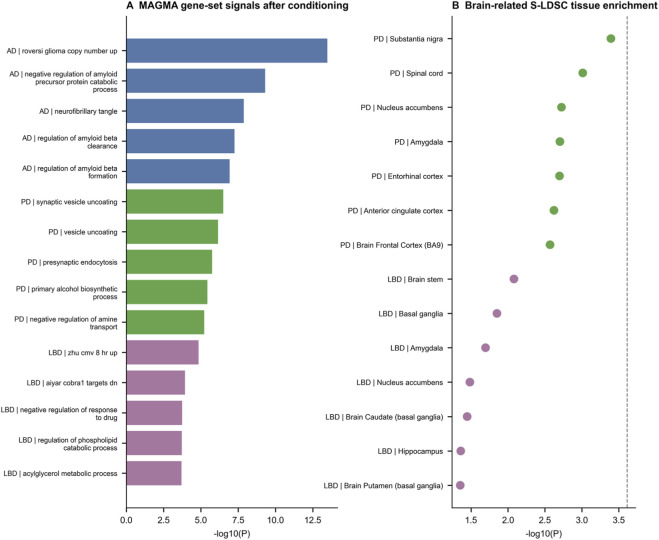
Functional enrichment after cross-disorder conditioning. **(A)** Top MAGMA gene-set signals in conditional AD, PD and LBD analyses, ranked by -log10(P). **(B)** Brain-related tissue enrichment from stratified LDSC in conditional PD and LBD analyses. The dashed line indicates the Bonferroni threshold for 205 tissue annotations.

S-LDSC showed the strongest brain-related enrichment for PD after conditioning, including substantia nigra, spinal cord, nucleus accumbens, amygdala, entorhinal cortex and anterior cingulate cortex (Figure 4B; Supplementary Table S8). LBD nominal enrichment involved brain-stem, basal-ganglia, amygdala and parietal-lobe annotations, whereas AD did not show Bonferroni-significant tissue enrichment in this framework.

MAGMA gene-set analyses contextualized retained loci (Figure 4A; Supplementary Table S9; Supplementary Figure 2). AD conditional signals involved amyloid-beta metabolism and clearance, neurofibrillary-tangle, lipid and immune-related terms. PD conditional signals involved synaptic vesicle uncoating, vesicle recycling, presynaptic endocytosis and glycosylceramide-related processes. LBD conditional gene-set results were weaker, consistent with its smaller GWAS sample size and reduced number of retained loci.

**FIGURE 5:**
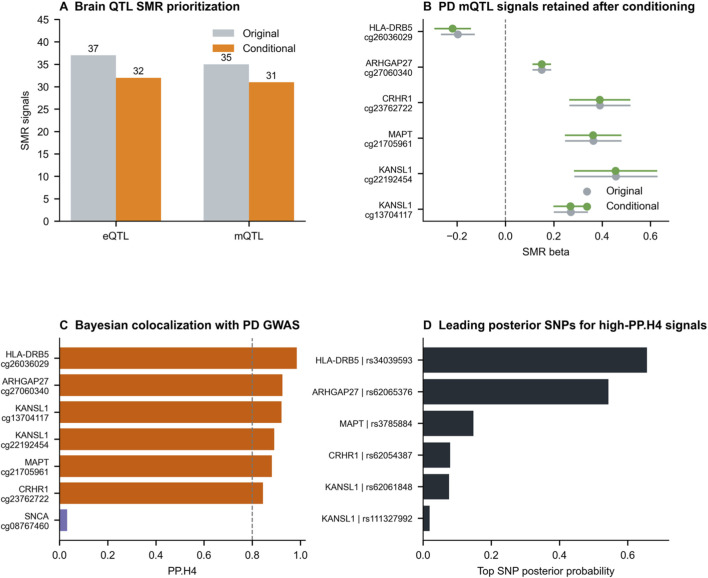
Brain QTL prioritization and Bayesian colocalization. **(A)** Counts of significant brain eQTL and mQTL SMR signals before and after conditioning. **(B)** PD mQTL SMR estimates before and after conditioning for retained methylation probes. Points indicate SMR beta estimates and horizontal lines indicate approximate 95% confidence intervals. **(C)** Posterior probability of a shared PD-GWAS and mQTL signal (PP.H4) from coloc. The dashed line marks PP.H4 = 0.80. **(D)** Highest SNP-level posterior probability for each high-PP.H4 PD colocalization analysis.

Brain QTL SMR analyses prioritized molecular associations at expression and methylation levels (Figure 5A; Supplementary Tables S10–S12). In the conditional analysis, 8 brain eQTL associations and 6 brain mQTL associations passed SMR and HEIDI filters. Conditional eQTL signals included AD-associated CR1 and PD-associated GPNMB, CD38, PLEKHM1, GAK and MAPT-region transcripts. Conditional mQTL signals included PD-associated HLA-DRB5, ARHGAP27, CRHR1, MAPT and KANSL1 probes. CR1, GAK and MAPT overlapped retained conditional GWAS loci.

The PD HLA-DRB5 mQTL signal became stronger after conditioning (adjusted SMR beta −0.218, SE 0.039; P = 1.51E-08; HEIDI P = 0.088), but the HLA region has extensive LD and complex immune genetics (Figrue 5B). Bayesian colocalization supported shared PD-GWAS and mQTL signals for HLA-DRB5 cg26036029 (36 harmonized SNPs; PP.H4 = 0.985), ARHGAP27 cg27060340 (78 SNPs; PP.H4 = 0.925), CRHR1 cg23762722 (167 SNPs; PP.H4 = 0.844), MAPT cg21705961 (134 SNPs; PP.H4 = 0.881), KANSL1 cg22192454 (130 SNPs; PP.H4 = 0.891) and KANSL1 cg13704117 (98 SNPs; PP.H4 = 0.922; Table 5; Figures 5C,D; Supplementary Tables S13–S15). PD-SNCA and LBD-SNCA mQTL tests instead supported distinct disease and mQTL association signals (PD PP.H3 = 0.969, PP.H4 = 0.031; LBD PP.H3 = 0.99999, PP.H4 = 1.95E-06).

**TABLE 5:** Bayesian colocalization of selected brain mQTL probes with disease GWAS signals.

Trait	Probe	Gene	nsnps	PP.H3	PP.H4
LBD	cg08767460	SNCA	154	1.000	0.000
PD	cg08767460	SNCA	144	0.969	0.031
PD	cg13704117	KANSL1	98	0.078	0.922
PD	cg21705961	MAPT	134	0.119	0.881
PD	cg22192454	KANSL1	130	0.109	0.891
PD	cg23762722	CRHR1	167	0.156	0.844
PD	cg26036029	HLA-DRB5	36	0.015	0.985
PD	cg27060340	ARHGAP27	78	0.075	0.925

## Discussion

This study links AD-PD clinical co-occurrence with cross-disorder conditional genetic analysis of AD, PD and LBD. The UK Biobank results show bidirectional clinical co-occurrence between AD and PD, while LDSC and GSMR support partial shared genetic liability among the three disorders. Conditional GWAS narrowed the association landscape to a smaller set of disease-predominant loci rather than a fully shared genetic architecture.

The retained loci are consistent with established disease biology while refining cross-disorder interpretation. AD retained loci involved CR1, BIN1, CLU, SPI1, MS4A, PICALM, ABCA7 and the APOE region, consistent with immune, lipid and amyloid-related pathways (Kunkle et al., 2019; Lambert et al., 2013; Karch and Goate, 2015). PD retained loci included SNCA, GBA-region, GAK/TMEM175, LRRK2, GPNMB, MAPT and RIT2-region signals, consistent with synuclein biology, lysosomal pathways, vesicle processes and immune-related mechanisms (Nalls et al., 2014; Nalls et al., 2019; Karch and Goate, 2015; Simon-Sanchez et al., 2009; Desikan et al., 2015; Tong and Chen, 2021; Dai et al., 2020; Wissemann et al., 2013; International Parkinson Disease Genomics Consortium, 2011; Little et al., 2017; Rana et al., 2019; Aarsland et al., 2017; Corces et al., 2020; Edwards et al., 2010; Moloney et al., 2018; Yao et al., 2021; Barbu et al., 2020; Soto-Beasley et al., 2020; Saeed, 2018; Witoelar et al., 2017). LBD retained signals at SNCA/MMRN1 and APOE/TOMM40/APOC1, consistent with its mixed synuclein and dementia-related genetic architecture and with clinical-pathological distinctions between LBD and Parkinson’s disease dementia (Guerreiro et al., 2018; Guerreiro et al., 2016; Jellinger and Korczyn, 2018; Chia et al., 2021; Jellinger, 2018; Hansen et al., 2021; Fritz et al., 2016).

Molecular integration prioritized regulatory signals within conditionally retained loci. The HLA-DRB5 methylation signal in PD was supported by SMR, HEIDI and colocalization, but this region has extended linkage disequilibrium and complex immune genetics. This result prioritizes the region and methylation probe for follow-up rather than assigning a definitive causal role to HLA-DRB5. The 17q21.31 PD signals involving ARHGAP27, CRHR1, MAPT and KANSL1 also showed high colocalization support, but the region contains extended haplotype structure and requires fine-scale validation.

The analysis has several limitations. First, the LBD GWAS was substantially smaller than the AD and PD GWAS, reducing power, instrument availability and stability for conditional estimates. Second, the LBD mtCOJO exposure instrument threshold was relaxed to P < 1E-06, so LBD conditional results should be viewed as exploratory. Third, summary-statistics-based conditioning cannot fully resolve linkage disequilibrium, sample overlap, cross-cohort heterogeneity or target-gene assignment. Fourth, UK Biobank AD and PD phenotypes were based on linked health-record first-occurrence fields, so diagnostic misclassification and surveillance bias remain possible. Fifth, SMR, HEIDI and colocalization prioritize shared genetic and molecular signals but do not establish causality; independent replication, fine-mapping and experimental validation remain necessary.

## Conclusion

AD and PD show bidirectional clinical co-occurrence in the UK Biobank, and AD, PD and LBD share part of their inherited liability. Cross-disorder conditional GWAS retained a smaller set of disease-predominant loci, and brain QTL integration prioritized regulatory signals including HLA-DRB5 and 17q21.31 methylation signals in PD. These findings provide a focused set of loci and molecular associations for independent genetic and functional validation.

## Data Availability

The original contributions presented in the study are publicly available. This data can be found in Zenodo with the DOI https://doi.org/10.5281/zenodo.20714624.
