# Supplementary material for: Disease-predominant loci across Alzheimer’s disease, Parkinson’s disease and Lewy body dementia: evidence from the UK Biobank prospective cohort, conditional GWAS and colocalization
Source: Front Genet. 2026 Jul 2;17:1865257. doi: 10.3389/fgene.2026.1865257 (PMC13372275; doi:10.3389/fgene.2026.1865257)

**Supplementary Figures**

**Supplementary Figure 1. Forest-plot summary of bidirectional IVW, MR-Egger and GSMR estimates before and after genetic conditioning.**
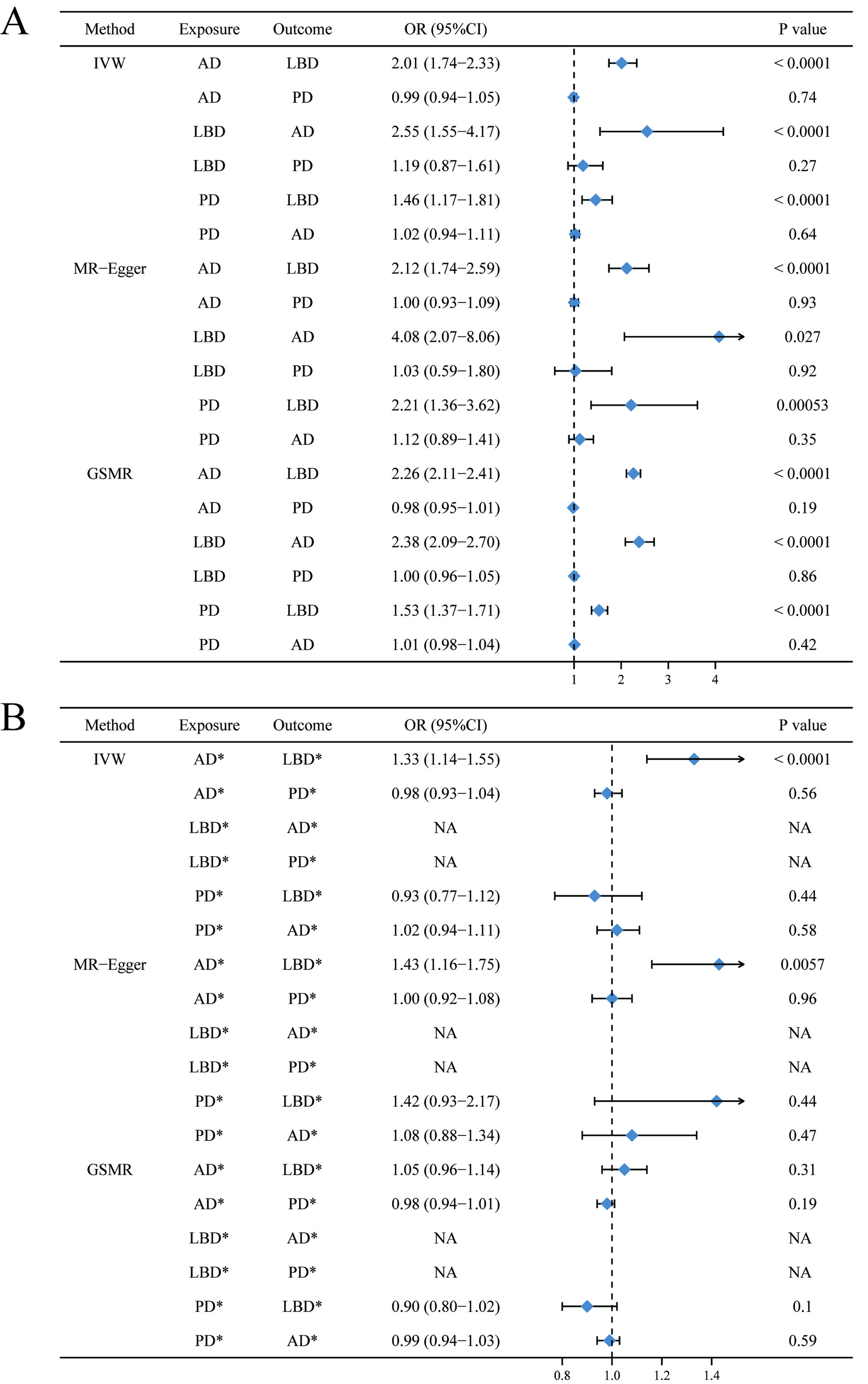


**Supplementary Figure 2. Gene-set enrichment bubble plots across original and conditional AD, PD and LBD GWAS analyses.**


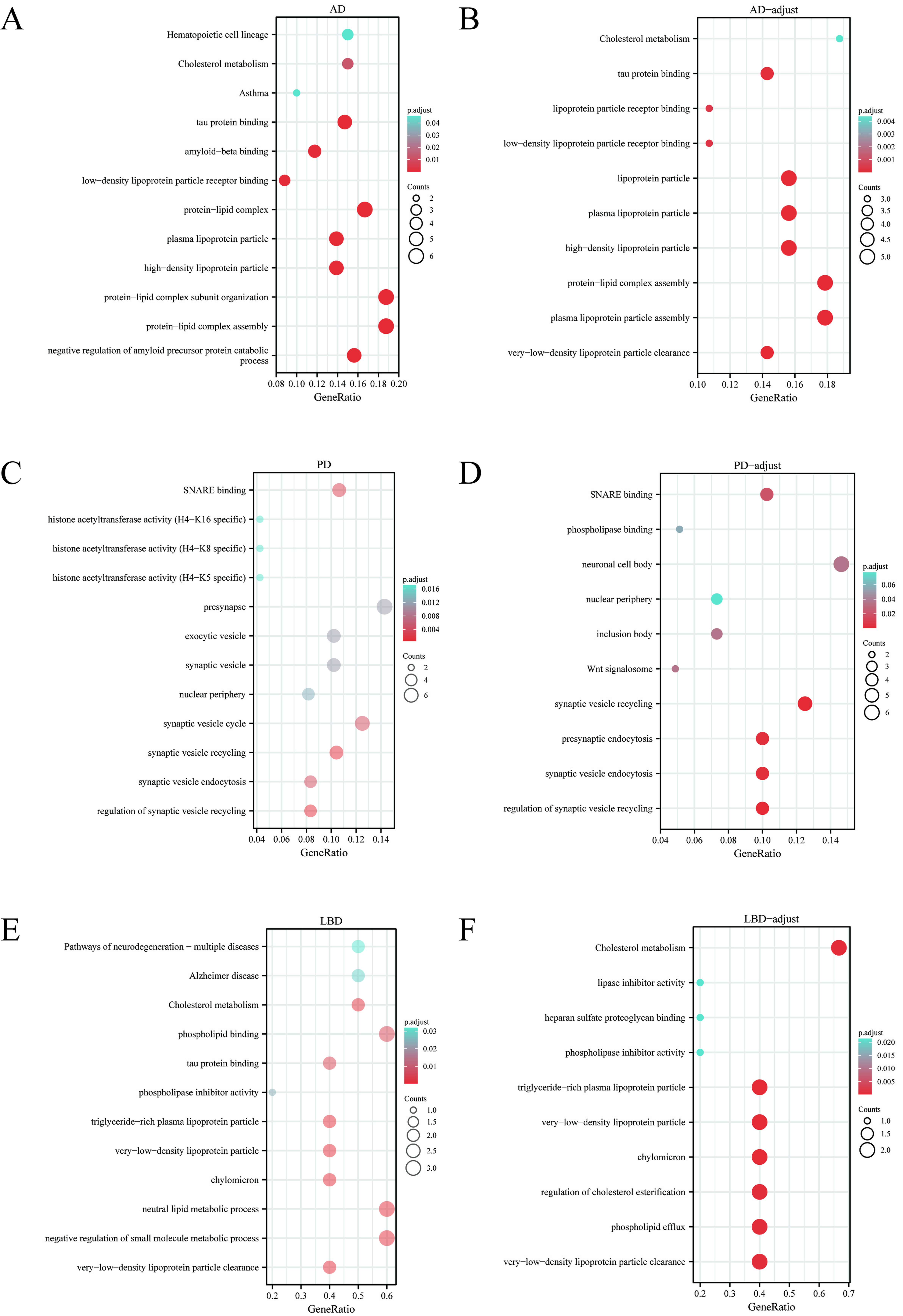

Supplement: Supplementary file 2 [file DataSheet1.docx]
